# EXPLORING INTERSECTIONALITY IN SPIRITUAL EXPERIENCES DURING DISEASE OF OLDER ADULTS AND CAREGIVERS

**DOI:** 10.1093/geroni/igad104.3418

**Published:** 2023-12-21

**Authors:** Sarah Won, Natalie Regier, Suratsawadee Kruahong, Changhwan Kim

**Affiliations:** Johns Hopkins University, Baltimore, Maryland, United States; Johns Hopkins University School of Nursing, Baltimore, Maryland, United States; Johns Hopkins University, Baltimore, Maryland, United States; Johns Hopkins University School of Nursing, Baltimore, Maryland, United States

## Abstract

Spirituality is known to be an important contributor to older adults’ and caregivers’ psychological and physical well-being. Despite this significance, their spiritual aspects are often overlooked within the context of treating and managing disease. Moreover, limited evidence exists regarding what specific intersectional identities of older adults and caregivers have the potential to cause disparities in their spiritual well-being. Therefore, the purpose of this study was to identify the extent to which intersectionality has been considered within the literature concerning factors associated with the spiritual aspects (defined as spiritual needs, spiritual support, and spiritual well-being) of older adults and their caregivers. Consequently, a five-stage integrative review was conducted using the protocol proposed by Whittemore and Knafl. This process yielded a total of 543 articles identified in PubMed, Embase, Cochrane, CINAHL, PsycINFO, and Web of Science in March 2023. Following the application of inclusion/exclusion criteria, 18 articles were selected for data extraction. Finally, four themes related to intersectional considerations were found: 1) The potential role of intersecting multiple identities in differences in spiritual experiences; 2) Older adults and caregivers, the pre-existing identities, that intensify the likelihood of intersectionality leading to disparities in spiritual experiences; 3) The importance of spiritual perspective in addressing challenges derived from the intersections of multiple identities; 4) Recommendations for professionals to consider potential intersectional factors in research and clinical contexts to improve inclusiveness. Findings highlight the necessity of adopting an intersectional lens within both research and clinical domains to prevent overlooking any resulting variations in spiritual experiences.

